# Sexual behaviors, HIV knowledge, HIV testing attitudes and recent HIV testing among female entertainment workers in Cambodia: A cross-sectional study

**DOI:** 10.1371/journal.pone.0198095

**Published:** 2018-07-02

**Authors:** Siyan Yi, Sovannary Tuot, Pheak Chhoun, Khuondyla Pal, Kolab Chhim, Chanrith Ngin, Carinne Brody

**Affiliations:** 1 KHANA Center for Population Health Research, Phnom Penh, Cambodia; 2 Public Health Program, College of Education and Health Sciences, Touro University California, Vallejo, CA, United States of America; University of New Mexico Health Sciences Center, UNITED STATES

## Abstract

**Background:**

In Cambodian context, female entertainment workers (FEWs) are young women working at establishments such as karaoke bars, restaurants, beer gardens or massage parlors. FEWs may sell sex to male patrons and are considered a high-risk group for HIV. This study aimed to identify factors associated with recent HIV testing among FEWs in Cambodia to inform future prevention activities.

**Methods:**

Data were collected in 2014 as part of the evaluation of a larger HIV prevention project. A two-stage cluster sampling method was used to select participants from Phnom Penh and Siem Reap for face-to-face interviews using a structured questionnaire. A logistic regression model was constructed to identify independent factors associated with recent HIV testing.

**Results:**

Data were collected from 667 FEWs with a mean age of 25.6 (SD = 5.5). Of total, 81.7% reported ever having had an HIV test, and 52.8% had at least one test in the past six months. After adjustment for other covariates, factors independently associated with recent HIV testing included living in Phnom Penh (AOR = 2.17, 95% CI = 1.43–3.28), having received HIV education in the past six months (AOR = 3.48, 95% CI = 2.35–5.15), disagreeing with a statement that ‘I would rather not know if I have HIV’ (AOR = 2.15, 95% CI = 1.41–3.30), agreeing with a statement that ‘getting tested for HIV helps people feel better’ (AOR = 0.32, 95% CI = 0.13–0.81) and not using a condom in the last sexual intercourse with a non-commercial partner (AOR = 0.48, 95% CI = 0.26–0.88).

**Conclusions:**

FEWs with greater knowledge and positive attitudes towards HIV testing got tested for HIV more frequently than those with lesser knowledge and less positive attitudes. These findings suggest that future interventions should focus on disseminating tailored health messages around testing practices as well as specific topics such as condom use with non-commercial partners.

## Introduction

In 2014, the HIV prevalence among the general adult population in Cambodia was 0.3%, reflecting a significant decline from the peak of 2.0% in 1998 [1,2]. Now, the HIV epidemic is confined mainly to high-risk groups such as sex workers, men who have sex with men (MSM), people who inject drugs and transgender women [3,4,5]. The reduction in HIV prevalence in the general population was attributed to the 100% condom use program that led to an increase in condom use, multi-sector programming that involved health workers, law enforcement officers, brothel owners and peer educators and increased access to voluntary confidential counseling and testing (VCCT) and antiretroviral therapy (ART) [6,7,8]. Cambodia was presented with a Millennium Development Goals (MDG) Award at the MDG Summit in 2010 for these efforts [9].

The passage and implementation of the 2008 ‘Law on Suppression of Human Trafficking and Sexual Exploitation,’ which banned brothel-based sex work has serious implications for HIV prevention [10]. The ‘brothel ban’ may have unintentionally created barriers to identifying and providing services to women who engage in commercial sex [11,12]. Cambodia has seen a significant decrease in brothel-based sex workers but an increase in sex workers at all types of entertainment venues, including karaoke bars, massage parlors and beer gardens [10,13].

Female entertainment workers (FEWs), with an estimated number of approximately 40,000 in the whole country, are now considered a high-risk group for HIV [14]. FEWs are young women who work at entertainment establishments, such as karaoke bars, beer gardens or massage parlors. FEWs may also sell sex to male patrons to supplement their income [15,16]. The prevalence of HIV among this group is estimated to be between 9.2%-13.9% [10, 11]. Reaching this population with prevention services, such as HIV testing, is a high priority in Cambodia. The Strategic Plan from 2016–2020 of the National Center for HIV/AIDS, Dermatology and STD (NCHADS) set a goal to reach at least 90% of HIV key populations, including FEWs, with an HIV test in every six months [17].

Entertainment venues have become important locations for prevention activities, including HIV education and community-based testing [13]. Still, the prevalence of lifetime HIV testing among FEWs in Cambodia has been found to be between 60–80% [11,18], and recent testing has been shown to be much lower, ranging from as low as 20.0% (in the past three months) [11] to 63.9% (in the past six months) [18]. Factors associated with HIV testing among sex workers in Asia include older age [18,19], higher educational attainment [19,20], longer duration working in entertainment venues [21], working in higher-income commercial sex venues [19], greater self-rated HIV knowledge [19,21], high perception of HIV risk [20], having a regular sexual partner [21], inconsistent condom use with husbands or lovers [22] and illegal drug use history [20,22]. There is currently no published information on whether or not these factors are associated with recent HIV testing for FEWs in Cambodia. In order to improve regular HIV testing rates among FEWs through tailored services, it is critically important to know factors affecting testing practices. This study aims to identify factors associated with recent HIV testing among FEWs in Cambodia including socio-demographic characteristics, sexual behaviors, HIV testing attitudes and HIV knowledge.

## Materials and methods

### Ethical statement

The National Ethics Committee for Health Research of the Ministry of Health, Cambodia approved this study (Reference no. 082NECHR). A written informed consent was obtained from each participant after they were made clear that participation in this study was voluntary, and they could refuse or discontinue their participation at any time. We protected privacy of the respondents by conducting the interviews at a private place, and no personal identifiers were collected in the questionnaires or field notes.

### Participants and sampling

This cross-sectional study was conducted in 2014 as part of the end-line survey to evaluate the impact of the Sustainable Action against HIV and AIDS in Communities (SAHACOM) Project. The five-year intervention was implemented by KHANA, the largest national non-governmental organization providing HIV prevention, care and support services in Cambodia. Reports related to the main survey have been published elsewhere [18, 23]. Venues from which study participants were selected for face-to-face interviews were randomly selected from a list of entertainment venues obtained from KHANA’s partners in Phnom Penh and Siem Reap. The number of FEWs in Phnom Penh and Siem Reap represents approximately 70% of the total FEW population in Cambodia [6]. The sample size was proportionally allocated to the number of FEWs in the two study sites.

We used a two-stage cluster sampling method to select participants from the two provinces. In the first stage, the probability-proportional-to-size sampling method was used to select entertainment venues from the list. At the second stage, a proportionate number of FEWs were randomly selected from each selected venue using a convenience sampling method. Inclusion criteria for the study included: (1) biologically female; (2) at least 18 years of age; (3) able to present themselves on the day of the interview; and (4) able to provide consent to participate in the study.

Research teams were trained for two days on the study methods, interview techniques, privacy assurance, confidentiality and quality control strategies. One day was allocated for questionnaire pretesting and revision after the training. Selected participants were approached by trained interviewers at the entertainment venues. Subjects were interviewed face-to-face after an informed consent has been obtained. The estimated time for each interview, including time for obtaining informed consent, was approximately 30 minutes.

### Questionnaire development and measurements

A structured questionnaire was first developed in English with inputs from experts working on HIV in Cambodia and then translated into Khmer, the national language of the country. The Khmer questionnaire was back-translated and pretested with a sample of 10 FEWs in Phnom Penh to ensure that the wording and content were culturally suitable and clearly understandable for the respondents. The questionnaire was finalized based on the feedback from the pretest.

Standardized tools were adapted from previous studies in the same population [24], the most recent Cambodia Demographic and Health Survey [25], as well as from other studies in Cambodia [26,27]. Demographic characteristics included age, marital status, years of education, types of establishment, average monthly income and duration working in as FEW as well as in the current place of employment. In addition, we also collected information on whether they had received HIV education and sexual and reproductive health information in the past six months and self-perception of their level of HIV risk compared to the general population.

To measure history of HIV testing, participants were asked about lifetime HIV testing, HIV testing in the past six months, place of most recent HIV test, person who advised them to get their most recent HIV test, if they received the result of their most recent HIV test and if they received counseling for their most recent HIV test. Those who had not been tested were asked the main reason for not taking the HIV test.

Several variables on sexual and reproductive health were measured including age at first sex, number of sexual partners in the past three months, number of partners with whom they had sexual intercourse in exchange for money or gifts (commercial partners) and those not in exchange for money or gift (non-commercial partners) in the past three months and condom use with both types of sexual partners in the past three months.

HIV testing attitudes were measured using five items adapted from a previous study on HIV testing and testing attitudes [28]. Two items reflected positive outcomes from testing, two assessed adverse outcomes and one item reflected HIV testing avoidance. The participants responded to each statement dichotomously, as either ‘‘agree” or ‘‘disagree.” To measure HIV knowledge, we used a 12-item test that was adapted by Kalichman and colleagues from an 18-item measure reported by Carey and Schroder [28,29] and reflected information about HIV transmission, condom use, and AIDS knowledge. ‘Don’t know’ responses were regarded as incorrect. The full report of the SAHACOM End-of-project Evaluation [23] can be found at: http://www.khana.org.kh/publicationimages/publican_pdf/RE018.pdf.

### Data analyses

Double data entry was performed using EpiData version 3 (Odense, Denmark). Two separate data entry clerks entered the data into separate data entry spreadsheets which were then compared; any discrepancies were decided by a third person using the paper questionnaire for reference. In univariate analyses, the history of HIV testing was calculated using proportions. In bivariate analyses, we used χ^2^ test, or Fisher’s exact test when sample sizes were smaller than five in one cell, for categorical variables, and Student’s *t*-test for continuous variables. A multivariable logistic regression model was then constructed to control for the effects of potential confounding factors. First, we included all variables associated with HIV testing in the past six months in bivariate analyses at a level of *p*< 0.05 in the model. All variables with a *p*-value> 0.05 were then removed from the model, and the model was refitted. The steps were repeated until all *p*-values of the remaining variables were <0.05 in the final model. Adjusted odds ratio (AOR) were obtained and presented with 95% confidence intervals (CI) and *p*-values. SPSS version 22 (IBM Corporation, New York, USA) was used for all statistical analyses.

## Results

### Socio-demographic characteristics and HIV testing history

Socio-demographic characteristics of the respondents are shown in **Table 1**. Data were collected from 667 FEWs, with a median age of 25.2 (range: 18 and 47) years. Of total, 78.4% were recruited in Phnom Penh. Almost half of the women were never married (41.4%) and worked in a karaoke bar (47.4%). Only 35.2% reported an average monthly income of >US$200. About half had worked as FEW for longer than 14 months and in the current venue for longer than eight months. In the past six months, 74.3% had received HIV education, and 64.8% had received sexual and reproductive health information. Interestingly, 52.1% of the women perceived that their HIV risk was lower compared to that of the general population.

**Table 1:** Comparisons of socio-demographic characteristics of FEWs who had and who had not been tested for HIV.

Socio-economic characteristics		Total (*n* = 667)	HIV testing in the past 6 months	
			Yes (*n* = 352)	OR (95% CI)^*^
Lived in Siem Reap		144 (21.6)	99 (68.8)	2.35 (1.59–3.47)
Age > 25 years		297 (44.5)	179 (60.3)	1.73 (1.27–2.35)
Marital Status				
	Never married	294 (41.4)	130 (44.2)	Reference
	Married	190 (28.5)	110 (57.9)	1.73 (1.20–2.51)
	Divorced, separated, or widowed	183 (27.4)	112 (61.2)	1.99 (1.37–2.90)
Completed formal education >7 years		411 (61.6)	205 (49.9)	0.74 (0.54–1.01)
Entertainment establishment				
	Karaoke bar	298 (47.4)	143 (48.0)	Reference
	Restaurant	201 (30.1)	117 (58.2)	0.69 (0.50–1.05)
	Massage parlor	84 (12.6)	47 (56.0)	1.38 (0.85–2.24)
	Beer garden	24 (3.6)	13 (54.2)	1.28 (0.56–2.95)
	Other	60 (9.0)	32 (53.3)	1.24 (0.71–2.16)
Average monthly income >$200		235 (35.2)	127 (54.0)	1.08 (0.79–1.49)
Worked as FEW for >14 months		330 (49.5)	195 (59.1)	1.66 (1.22–2.25)
Worked in current venue > 8 months		316 (47.4)	190 (60.1)	1.76 (1.29–2.39)
Received HIV education in the past 6 months		495 (74.3)	300 (85.2)	3.52 (2.43–5.11)
Received SRH information in the past 6 months		432 (64.8)	254 (58.8)	2.00 (1.45–2.75)
Self-regarded HIV risk compared to the general population				
	Higher	154 (23.1)	86 (55.8)	Reference
	Same	93 (13.9)	55 (59.1)	1.14 (0.68–1.93)
	Lower	339 (50.8)	175 (51.6)	0.84 (0.58–1.24)
	Don’t know	81 (12.1)	36 (44.4)	0.63 (0.37–1.09)

Abbreviations: CI, confidence interval; FEWs, female entertainment workers; HIV, human immunodeficiency virus; OR, odds ratio; SRH, sexual and reproductive health.

*Chi-square test (or Fisher’s exact test when a cell count was smaller than one) was used.

Of all participants, 81.7% reported ever having had an HIV test, and 52.8% reported having had an HIV test in the past six months. The majority of them received the result and counseling for their most recent test. For those who had never been tested, the main reasons for not getting tested included self-perception of low HIV risk (71.1%), fear of the test (11.7%), no information about where to get tested (5.5%), fear of the positive result (3.1%) or other non-specified reasons (8.6%).

As also shown in **Table 1**, the proportion of FEWs who had been tested for HIV in the past six months was significantly higher among FEWs living in Phnom Penh compared to that among FEWs living in Siem Reap (OR = 2.35, 95% CI = 1.59–3.47) and among FEWs who were older than 25 compared to those aged 25 or younger (OR = 1.73, 95% CI = 1.27–2.35). The proportion was significantly higher among FEWs who were married (OR = 1.73, 95% CI = 1.20–2.51) and divorced, separated or widowed (OR = 1.99, 95% CI = 1.37–2.90) compared to that among never married women. The proportion was significantly higher among FEWs who had worked as FEW (OR = 1.66, 95% CI = 1.22–2.25) and in the current venue (OR = 1.76, 95% CI = 1.29–2.39) for a longer duration and those who had received HIV education (OR = 3.52, 95% CI = 2.43–5.11) and sexual and reproductive health information (OR = 2.00, 95% CI = 1.45–2.75) in the past six months compared to those who had not.

### Sexual and reproductive health

Sexual and reproductive health and behaviors of the respondents are shown in **Table 2**. About half of the women reported having their sexual intercourse when they were older than 19 years old, and about one-third reported having more than two sexual partners in the past three months. Of those who reported having sexual intercourse with non-commercial partners, 38.2% reported using a condom in the last intercourse. The reported rate of consistent condom use with commercial partners was considerably high at 78.7%. About two-thirds responded that they could find condoms whenever they needed. More than one in five reported having been diagnosed with a sexually transmitted infection (STIs) in the past three months.

**Table 2:** Comparisons of sexual and reproductive health and behaviors among FEWs who had and who had not been recently tested for HIV.

Sexual behaviors in the past 3 months	Total (*n* = 667)	HIV testing in the past 6 months	
		Yes (*n* = 352)	OR (95% CI)*
Age at first sex >19 years	334 (50.1)	198 (59.3)	1.69 (1.25–2.30)
Number of sex partners >2	226 (33.9)	114 (50.4)	0.87 (0.63–1.20)
Used condom at last sex with a non-commercial partner	78 (38.2)	38 (48.7)	0.53 (0.30–0.94)
Always used condoms with commercial partners	100 (78.7)	58 (58.0)	0.91 (0.38–2.13)
Able to find condoms as needed	426 (63.9)	253 (59.4)	2.10 (1.52–2.89)
Diagnosed with an STI	150 (22.5)	86 (57.3)	1.27 (0.88–1.84)

Abbreviations: CI, confidence interval; FEWs, female entertainment workers; HIV, human immunodeficiency virus; OR, odds ratio; STI, sexually transmitted infections.

*Chi-square test (or Fisher’s exact test when a cell count was smaller than one) was used.

**Table 2** also shows that the proportion of FEWs who had been tested for HIV in the past six months was significantly higher among those who reported having their sexual intercourse when they were older than 19 years old (OR = 1.69, 95% CI = 1.25–2.30), and those who reported that they were able to find condoms whenever they needed (OR = 2.10, 95% CI = 1.52–2.89). This proportion was significantly lower among FEWs who reported using a condom at the last sexual intercourse with a non-commercial partner compared to those who did not (OR = 0.53, 95% CI = 0.30–0.94).

### HIV testing attitudes

HIV testing attitudes are presented in **Table 3.** Of total, FEWs agreed that getting tested for HIV helps people feel better (95.8%); getting tested for HIV helps prevent people from getting HIV (86.3%); people in their life would leave them if they had HIV (44.6%); people who test HIV positive should hide it from others (31.5%) and they would rather not know if they have HIV (19.5%). The proportion of FEWs who had been tested for HIV in the past six months was significantly higher among those who agreed that getting tested for HIV helps people feel better (OR = 0.34, 95% CI = 0.15–0.79) and lower among those who agreed that they would rather not know if they have HIV (OR = 2.41, 95% CI = 1.62–3.59).

**Table 3:** Comparisons of HIV testing attitudes among FEWs who had and who had not been tested for HIV.

HIV testing attitudes items	Total (*n* = 667)	HIV testing in the past 6 months	
		Yes (*n* = 352)	OR (95% CI) ^*^
Getting tested for HIV helps people feel better	637 (95.8)	344 (54.0)	0.34 (0.15–0.79)
Getting tested for HIV helps people from getting HIV	575 (86.3)	308 (53.6)	0.81 (0.52–1.26)
People in my life would leave me if I had HIV	297 (44.6)	159 (53.5)	0.95 (0.70–1.29)
People who test HIV positive should hide it from others	210 (31.5)	244 (51.4)	1.08 (0.78–1.50)
I would rather not know if I have HIV	130 (19.5)	46 (35.4)	2.41 (1.62–3.59)

Abbreviations: CI, confidence interval; FEWs, female entertainment workers; HIV, human immunodeficiency virus; OR, odds ratio.

Values are number (%) of respondents who agreed with the HIV testing items.

*Chi-square test (or Fisher’s exact test when a cell count was smaller than one) was used.

### HIV knowledge

As shown in Table 4, the majority of FEWs answered correctly to most of the knowledge questions, although room for improvement remained. The proportion of FEWs who had been tested for HIV in the past six months was significantly higher among those who responded correctly to the following items: ‘Can a person get AIDS by sharing a bathroom with someone with HIV?’ (OR = 1.82, 95% CI = 1.06–3.12); ‘Does washing after sex help protect against HIV?’ (OR = 1.68, 95% CI = 1.20–2.35) and ‘Is there a cure for AIDS?’ (OR = 1.59, 95% CI = 1.03–2.44).

**Table 4:** Correct responses to HIV knowledge items among FEWs who had and who had not been tested for HIV.

HIV knowledge items	Total (*n* = 667)	HIV testing in the past 6 months	
		Yes (*n* = 352)	OR (95% CI) ^*^
Is AIDS spread by kissing? (No)^†^	619 (92.9)	329 (53.2)	1.29 (0.71–2.34)
Can a person get AIDS by sharing bathrooms with someone with HIV? (No)	606 (90.9)	328 (54.1)	1.82 (1.06–3.12)
Can men give HIV to women? (Yes)	645 (96.7)	342 (53.0)	1.35 (0.58–3.18)
Can women give HIV to men? (Yes)	641 (96.1)	343 (53.5)	2.17 (0.96–4.95)
Must a person have many different partners to get HIV? (No)	125 (18.8)	64 (51.2)	0.93 (0.63–1.38)
Can you get HIV by touching someone with HIV? (No)	611 (91.6)	323 (52.9)	1.04 (0.60–1.81)
Does washing after sex help protect against HIV? (No)	469 (70.4)	265 (56.5)	1.68 (1.20–2.35)
Is AIDS caused by spirits/supernatural forces? (No)	609 (91.7)	323 (53.0)	1.17 (0.67–2.03)
Can a pregnant woman give AIDS to her baby? (Yes)	432 (64.9)	224 (51.9)	0.91 (0.66–1.25)
Can a person get rid of AIDS by having sex with a virgin? (No)	441 (66.1)	238 (54.0)	1.15 (0.84–1.59)
Is HIV the virus that causes AIDS? (Yes)	384 (57.6)	208 (54.2)	1.14 (0.84–1.55)
Is there a cure for AIDS? (No)	567 (85.0)	309 (54.5)	1.59 (1.03–2.44)

Abbreviations: AIDS, acquired immune deficiency syndrome; CI, confidence interval; FEWs, female entertainment workers; HIV, human immunodeficiency virus; OR, odds ratio.

Values are number (%) of respondents who answered correctly to the HIV knowledge items.

*Chi-square test (or Fisher’s exact test when a cell count was smaller than one) was used.

^†^Correct responses are shown in parentheses.

### Factors associated with HIV testing

**Table 5** presents factors associated with recent HIV testing among FEWs in multivariate logistic regression model. After adjustment for other covariates, factors independently associated with recent HIV testing included living in Phnom Penh (AOR = 2.17, 95% CI = 1.43–3.28), having received HIV education in the past six months (AOR = 3.48, 95% CI = 2.35–5.15), disagreeing with a statement that ‘I would rather not know if I have HIV’ (AOR = 2.15, 95% CI = 1.41–3.30), agreeing with a statement that ‘getting tested for HIV helps people feel better’ (AOR = 0.32, 95% CI = 0.13–0.81), and not using a condom in the last sexual intercourse with a non-commercial partner (AOR = 0.48, 95% CI = 0.26–0.88).

**Table 5:** Factors associated with recent HIV testing among FEWs in multivariable logistic regression model (*n* = 667).

Variables in the final model*		HIV testing in the past 6 months	
		AOR (95% CI)	*p*-value
Study sites			
	Phnom Penh	Reference	
	Siem Reap	2.17 (1.43–3.28)	<0.001
Received any forms of HIV education in the past 6 months			
	No	Reference	
	Yes	3.48 (2.35–5.15)	<0.001
Condom use in the last sex with a non-commercial partner			
	No	Reference	
	Yes	0.48 (0.26–0.88)	0.02
Getting tested for HIV helps people feel better			
	Agree	Reference	
	Disagree	0.32 (0.13–0.81)	0.02
I would rather not know if I have HIV			
	Agree	Reference	
	Disagree	2.15 (1.41–3.30)	<0.001

Abbreviations: AOR, adjusted odds ratio; CI, confidence interval; FEWs, female entertainment workers.

*Adjusted for age, marital status, years of working as FEW, age at first sexual intercourse, access to condoms, and HIV knowledge items significantly associated with recent HIV testing in bivariate analyses.

## Discussion

About half of FEWs (52.3%) in our study had been tested for HIV in the past six months, and 81.7% had been tested in lifetime. Compared to past studies with similar populations, these rates may represent an improvement. In 2009, a study in Cambodia found that sex workers had a lifetime prevalence of HIV testing of 79.7%, and three months testing prevalence of 22.2% [11]. A situational analysis of FEWs in Cambodia in 2012 found that four out of 41 participants had ever been tested for HIV, although this study was not designed to be representative of FEWs in the cities where the study was conducted [13]. In China, a study of female sex workers in the southeast found that 48% had ever been tested for HIV [19]. It is difficult to compare HIV testing rates of FEWs in Cambodia to other studies because many focus exclusively on female sex workers or measure testing rates at different time intervals. Nevertheless, our findings suggest that the recent testing rate for FEWs is much lower than the 90% goal of recent HIV testing (in every six months) that the nation sets for key populations such as FEWs. This may explain HIV testing fatigue among the women. FEWs may not want to get tested for HIV frequently if they are not aware of the recommendation of testing frequency for individuals who engage in high-risk behaviors. Moreover, despite their engagement in HIV risks, a large proportion of FEWs in this study did not perceive themselves as being at elevated risks.

Living in Phnom Penh, versus Siem Reap, was significantly associated with recent HIV testing. This is not surprising given that Phnom Penh is a densely packed city, and information dissemination as well as the penetration of community-based testing and VCCT sites is high. The higher proportion of FEWs in Phnom Penh receiving HIV testing in the past six months may be due to the fact that there were fewer testing sites and only one implementing partner who provided community-based finger prick testing to FEWs in Siem Reap during the study period, while there were more testing sites and two partners conducting community-based finger prick testing in Phnom Penh. Siem Reap is a high-risk area, and as other recent studies have suggested, this region could benefit from increased resources for HIV prevention activities [30].

Our findings suggest that receiving any form of HIV education in the past six months was associated with recent HIV testing. According to a recent study of MSM in Cambodia, the odds of having received any form of HIV education in the past six months and HIV testing was similar to FEWs in this study (FEWS = 3.48 vs. MSM = 3.97) [16,30]. This finding reinforces the effectiveness of high-quality HIV education and outreach programming.

Reported condom use at last sex with a non-commercial partner was negatively associated with recent HIV testing suggesting that FEWs who were more likely to have had a recent HIV test were less likely to have used condoms with their non-commercial partners. A study of female sex workers in Senegal reported a similar relationship between condom use with regular partners and history of HIV testing [31]. This is a concerning finding given that only 38.2% of all FEWs and 31.9% of FEWs that have been tested in the last six months used condoms at last sex with non-commercial partners. Getting tested and finding out that they are HIV negative may change FEWs perceptions of their risk and therefore may result in an increase in risky behaviors, such as decreased condom use with non-commercial partners.

In addition, this relationship may be specific to females and may represent their ability to negotiate condom use in their relationships. The opposite relationship was found in a study in Cambodia on MSM between condom use with boyfriends and recent HIV testing. Those respondents who had been recently tested for HIV were more likely to have used condoms at last intercourse with a boyfriend [18,32].

In our study, positive attitudes towards HIV testing were associated with recent HIV testing. Specifically, agreeing that ‘Getting tested for HIV helps people feel better’ and disagreeing that ‘I would rather not know if I have HIV’ were significantly associated with recent testing. A study from South Africa also found that positive attitudes are associated with HIV testing in female sex workers [28]. Despite this relationship, we found that those who had not been tested recently still exhibited highly positive attitudes towards HIV testing. For example, 93.6% of those who have not been recently tested agreed that getting tested for HIV helps people feel better, and 85.0% agreed that getting tested for HIV helps people from getting HIV. This may indicate that positive attitudes may not always lead to increased testing and that other structural or societal barriers may be preventing FEWs from getting testing frequently.

Important limitations of this study include the representativeness of the sample, the sampling strategy and the validity of the measures. First, only two provinces where the SAHACOM project had been implemented for FEWs were included in the study. Thus, the findings may not reflect FEWs in other areas of Cambodia. Future research should include FEWs in other areas, especially those not covered by the project. Second, the data for this paper were derived from an impact evaluation of the SAHACOM project. Thus, the sampling method was not necessarily designed for this analysis. Third, the self-reported measures may have caused under-reporting and over-reporting biases. Lastly, causal associations among the variables could not be established because of the cross-sectional nature of the study.

## Conclusions

FEWs with greater knowledge about and positive attitudes towards HIV testing got tested for HIV more frequently than those with lesser knowledge and less positive attitudes. These findings suggest that future interventions should focus on increasing outreach to groups that have not yet been reached with HIV education. In particular, supporting changes in beliefs and norms around healthy sexual practices could have an important role to play in getting to 90% coverage for recent testing. Some methods may include behavioral models that offer tailored health behavior messages around HIV testing practices as well as specific topics such as condom use with non-commercial partners. In addition, efforts to reduce stigma around HIV testing and knowing one’s HIV status may improve attitudes towards HIV testing and, therefore, may be important areas for future programming for FEWs in Cambodia.
